# The long developmental trajectory of body representation plasticity following tool use

**DOI:** 10.1038/s41598-020-79476-8

**Published:** 2021-01-12

**Authors:** Marie Martel, Livio Finos, Eric Koun, Alessandro Farnè, Alice Catherine Roy

**Affiliations:** 1 Laboratoire Dynamique Du Langage, CNRS UMR5596, Lyon, France; 2 University of Lyon II, Lyon, France; 3 Integrative Multisensory Perception Action and Cognition Team - ImpAct, Lyon Neuroscience Research Center, INSERM U1028, CNRS U5292, Lyon, France; 4 University UCBL Lyon 1, University of Lyon, Villeurbanne, France; 5 Department of Statistical Sciences, University of Padua, Padua, Italy; 6 Hospices Civils de Lyon, Mouvement Et Handicap and Neuro-Immersion, Lyon, France; 7 Center for Mind/Brain Sciences (CIMeC), University of Trento, Trento, Italy; 8 Present Address: Department of Psychology, Royal Holloway University of London, Egham Hill, Surrey, Egham, TW20 0EX UK

**Keywords:** Sensory processing, Human behaviour

## Abstract

Humans evolution is distinctly characterized by their exquisite mastery of tools, allowing them to shape their environment in more elaborate ways compared to other species. This ability is present ever since infancy and most theories indicate that children become proficient with tool use very early. In adults, tool use has been shown to plastically modify metric aspects of the arm representation, as indexed by changes in movement kinematics. To date, whether and when the plastic capability of updating the body representation develops during childhood remains unknown. This question is particularly important since body representation plasticity could be impacted by the fact that the human body takes years to achieve a stable metric configuration. Here we assessed the kinematics of 90 young participants (8–21 years old) required to reach for an object before and after tool use, as a function of their pubertal development. Results revealed that tool incorporation, as indexed by the adult typical kinematic pattern, develops very slowly and displays a u-shaped developmental trajectory. From early to mid puberty, the changes in kinematics following tool use seem to reflect a shortened arm representation, opposite to what was previously reported in adults. This pattern starts reversing after mid puberty, which is characterized by the lack of any kinematics change following tool use. The typical adult-like pattern emerges only at late puberty, when body size is stable. These findings reveal the complex dynamics of tool incorporation across development, possibly indexing the transition from a vision-based to a proprioception-based body representation plasticity.

## Introduction

To efficiently control our actions, whether performed with our hands or handheld tools, the brain needs to encode, update and store properties of the effector, including its size and shape, as well as its location. This “state of the body” is combined with the “state of the world” (e.g., the object to grasp) to build an appropriate motor plan^1–3^. The body state representation, also known as body schema, is highly plastic. Several studies converged in showing that using a tool modifies the effector representation, as indexed by changes in the kinematics of subsequent free-hand movements. Typically, after using a 40 cm long mechanical grabber, adult participants (in their mid-twenties) display longer latencies and smaller peaks in the reaching phase of their hand movements^4–8^. In other words, after tool use, participants move as if their arm is longer^8^ and these changes in their kinematics have been taken as a motor signature of tool incorporation. In adults, this plasticity is fast: a few minutes of tool use are enough to update the arm representation, making these changes in motor control observable (see, for review^9–12^).

From a developmental perspective, research agrees that humans are tool-users since their infancy^13–16^. Percussive hammering for instance seems to develop from infants’ own manual behaviour to allow proper use of a hammer by 3 years of age, distinguishing young children from nonhuman primates^17–19^. The ability to use a rake has been observed in infants around 18 months: in presence of a distant toy, they successfully use the rake to retrieve the toy by learning from their errors and adults’ demonstration^20–22^. Progression in such ability would be closely linked to improvement in vision, tool use following the perception–action loop similarly as any manual action^23–25^. Improvement in motor capabilities such as perception of affordances would tune improvement in tool use^26,27^, thus being tightly associated with multisensory integration. Later on, children become proficient tool users: by 6 years of age they can make reliable reachability judgements either with their arm or with a tool^28–31^, similarly to adults^32^. However, the developmental trajectory of the plastic capability of incorporating tools remains unknown and no study has tested whether tool use modifies the effector representation in children. This question is particularly important when considering that the human body and its representation undergo enormous changes, taking years to achieve a stable metric configuration^33,34^.

How does body representation deal with body growth to achieve skilful motor control? Besides the need to take their growing body size into account, children face the challenge of a developing multisensory integration, with immature use and weighting of vision and proprioception for instance, and immature motor control during the first decade of their life (e.g.^35–45^). Updating the body representation with the increasing metric of effectors implies extracting their current estimated size and weight, especially challenging during adolescence, a period of critical changes in body morphology. Indeed, while the rate of growth changes is almost constant during childhood and thus possibly simpler to cope with, it increases sharply during the growth spurt, a developmental stage usually related to a transitory clumsiness (^46^ but see^47^). As the brain computes current “state of the body” when programming a movement^2^, one could argue that estimates are more accurate in the case of a constant rate of growth, while growth spurt would be more difficult to track, particularly given multisensory integration is still under development. Velocity in physical growth has been shown, indeed, to be negatively correlated to motor competence^48^. For instance, 8-year-old children can optimally integrate visual and proprioceptive cues to localize their hand during movement^49,50^. Older children though, around 12 years old, display impoverished integrative performance, this decreased ability being attributed to the beginning of puberty^49^. In the same vein, Visser and Geuze^51^ observed that kinesthetic acuity improves from the age of 5–12 and from 14 to 16, but not from 11.5 to 14, in correspondence to the beginning of the adolescence growth spurt. In addition, temporary changes in proprioceptive integration have been highlighted in upper limb postural control. Anticipatory postural adjustments require a maturation period, similarly to voluntary control^43^, not being fully achieved before 8 years of age^52–55^. Interestingly, 11–16 years old adolescents do no perform better than younger kids^56^, actually failing to use proprioceptive information to improve their postural control^57^. These findings suggested that “adolescence might constitute a transient period of proprioceptive neglect in sensory integration of postural control”^57,58^. Thus, growth spurt may impact proprioceptive integration, which is necessary both for appropriate postural control of the upper limb^35^ and for tool use plastic changes in kinematics to manifest^8,59^.

Is tool-induced body representation plasticity present in children as it is in adults? If adolescence is a critical node in the developmental trajectory for the refinement of skillful motor control of the arms (slow plasticity), it might be so also when considering the plastic changes in kinematics induced by tool use (fast plasticity). To test this hypothesis, here we used a tool-use paradigm, well-established in adults, to investigate the developmental trajectory of tool-induced body representation plasticity in 90 participants (7.5- to 21.5-year-old) as a function of their adolescence (puberty score). The typical adult pattern (participants in their mid-twenties) shows increased latencies and reduced amplitudes of free-hand movements after tool use^5,8^. Here, we investigated if and when this pattern is observable in children, adolescents and young adults, contrasting two predictions: if tool use incorporation embraces the growing body dimensions smoothly, we should find the motor signature of tool use throughout the tested period. Alternatively, if growing spurt alters this ability, as suggested by the literature reviewed above, we should not observe the typical kinematics changes after tool use thorough all the developmental steps, and they should be particularly absent during adolescence.

## Material and methods

### Participants

Ninety typically developing children, adolescents and young adults (44 males; 6 left handed according to the Edinburgh inventory^60^; mean age ± SD: 13.95 ± 3.64; range: 7.5–21.5) were recruited from different local schools and universities to participate in this study. Data collection stopped at the end of the school year. Minimal age of recruitment was 7 years, to ensure that all participants would be able to anticipate the action goal in a similar fashion^43^. As for the maximal age, we enrolled the youngest participants possible who scored 20/20 at the puberty questionnaire (see below), which lead to recruit beyond 18 years old. Table 1 summarizes demographic data for each puberty score. All participants (their parents or guardians if under 18 years of age) gave written informed consent to participate to the study, which was approved by the French ethics committee (*Comité de Protection des Personnes* CPP Sud-Est II) and conformed to the Helsinki declaration. All were naïve to the purpose of the study, and had normal or corrected-to-normal vision, with no known neurological disorders, learning disabilities or delayed psychomotor acquisition according to their own report, or their parents’ one for the underage participants. None of the participants was born preterm. After completion of the tasks, each adult participant received a monetary compensation, while children and adolescents received a board game.

**Table 1:** Main characteristics of the participants for each puberty score.

Puberty score	Sample	Sex	Age (mean year)	Height (mean cm)	Forearm length (mean cm)	Tool length (cm)	Gesture imitation (score)
5	4	1F/3M	9.7 (8.1–11.1)	144 (135–165)	19.1 (18–20)	32 (n = 3) 40 (n = 1)	49.7 (43–61)
6	6	2F/4M	9.6 (7.5–11.9)	133 (118–140)	19.8 (16–22)	25 (n = 1) 32 (n = 5)	52.7 (39–60)
7	7	4F/3M	10.0 (7.6–13.8)	140 (127–150)	20.2 (18–23)	32 (n = 4) 40 (n = 3)	54.1 (39–66)
8	7	2F/5M	11.0 (9.3–12.9)	144 (135–158)	21.1 (19–24)	32 (n = 5) 40 (n = 2)	54.7 (48–61)
9	8	3F/5M	12.1 (9.2–15.0)	153 (138.5–171)	22.2 (20–26)	32 (n = 2) 40 (n = 6)	57.3 (50–68)
10	4	1F/3M	11.5 (9.0–14.4)	143 (136–154)	21.3 (19.5–23.5)	32 (n = 3) 40 (n = 1)	58.3 (48–66)
11	5	3F/2M	13.5 (11.2–14.7)	160 (144–175)	23.6 (22–25)	32 (n = 1) 40 (n = 4)	56.2 (49–66)
12	3	2F/1M	13.1 (12.5–13.5)	160 (155–163)	24.5 (23.5–26)	40	56.3 (53–62)
13	2	2F	12.6 (11.8–13.5)	158 (155–160)	24 (23–25)	40	60.0 (57–63)
14	4	1F/3M	14.8 (12.7–17.8)	168 (157–177)	24.7 (22–26)	40	60.5 (52–68)
15	8	5F/3M	14.6 (13.2–16.7)	167 (157–183)	24.7 (22–29)	40	62.1 (54–67)
16	3	2F/1M	15.9 (15.0–18.4)	172 (168–179)	25.5 (23.5–28)	40	59.0 (45–68)
17	6	4F/2M	15.3 (13.1–18.0)	171 (158–187)	25.3 (22–28)	40	48.0 (35–60)
18	3	2F/1M	15.7 (13.4–18.6)	165 (153–172)	23.5 (23–24.5)	40	60.0 (54–65)
19	11	9F/2M	17.8 (15.8–20.8)	165 (153–185)	24.4 (20.5–28)	40	59.9 (52–69)
20	9	3F/6M	19.8 (16.5–21.5)	172 (159–180)	25 (22–27)	40	61.5 (56–68)
Total	90	46F/44M	13.6 (7.5–21.5)	157 (118–187)	23 (16–29)	25 (n = 1) 32 (n = 23) 40 (n = 66)	56.9 (35–69)

### Apparatus and procedures

To account for changes in arm motor control induced by tool use, we used a well-established paradigm^5,8,11^: free-hand reach-to-grasp movements are performed before and after reach-to-grasp tool movements. The kinematics of free-hand movements are then compared (pre-post) to assess the consequences of tool use on free-hand movements. As a control for the specificity of tool use effects on body representation for action (body schema), participants performed the Arm Length Estimation task, which assesses the explicit, subjective estimate of the arm length (body image), typically left unchanged in adults^8^. Participants were comfortably seated in front of a table, on an adjustable chair, their dominant hand closed in a pinch-shaped grip on a starting switch. The target object was a wooden parallelepiped (10 × 2, 5 × 5 cm, weighting 96 g) placed on the table at a distance of 35 cm along the sagittal axis, in line with participants’ right shoulder (or left shoulder for left-handed participants). Importantly, as in previous studies using the same paradigm^4–8^, the target object was always located inside the arm reaching space, thus preventing the potential confounding effects of tool use in different sectors of space (reachable vs. non-reachable space). Once reached a comfortable position, the chair was fixed to keep the distance from the table constant, but could rotate to adapt to the different tasks.

**Figure 1:**
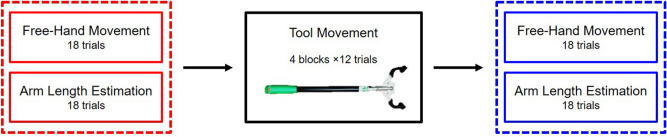
Experiment timeline. The tool use session included 4 blocks of 12 trials. Pre and post tool use sessions consisted of 18 free-hand reach-to-grasp movements and arm length estimation trials. The Free-Hand Movement and the Arm Length Estimation tasks were run by blocks in a counterbalanced order across participants.

The experiment was composed of three sessions: a pre- and post-tool use session, separated by a tool use session (Fig. 1).

The pre and post tool-use sessions included the two tasks (Free-Hand Movement and Arm Length Estimation), counterbalanced across participants. In the Free-Hand Movement task (18 trials), participants had to reach, grasp and lift an object with their dominant hand, at their natural speed. Then, participants put the object down and brought their hand back to the starting position on the switch, waiting for an auditory signal to start the next trial. The reaching component of the movement was characterized by the kinematic parameters of the wrist, while the grip component was characterized by the thumb and index displacement. For each movement, the following kinematic parameters were extracted and analysed off-line: latencies and amplitudes of wrist acceleration, wrist velocity and wrist deceleration (reaching component), latency and amplitude of the maximum thumb-index distance (hereafter MGA for Maximum Grip Aperture) (grasping component). For each movement we measured the overall movement time as the time between the beginning of the movement (velocity ≥ 10 mm/s after switch release) and stabilized grasp on the object (before any lifting). In the Arm Length Estimation task (18 trials), participants were blindfolded and had to slide their dominant index finger horizontally on the surface of the table from the starting position to a final point for a distance they estimated to be equal to the distance between their wrist and elbow^8^. This distance was extracted and considered as the participants’ perceived arm length. These body parts were named and touched by the experimenter while giving the tasks instructions to the participant. The instructions were given once blindfolded to ensure that participants would not try to measure their arm before starting, or to use any strategy, which could bias their estimation.

The tool use session comprised four blocks of 12 trials each (48 trials in total), during which participants had to squeeze the tool handle to hold the tool prongs in a pinch grip position on the same starting switch, and then reach, grasp and lift the same target object using the tool. Each participant underwent a short practice tool use session at the beginning of the tool session. Extracted parameters were the same as in the Free-Hand Movement task, except that they involved kinematics of the tool instead of the hand (see section Kinematic Recording System for details on the markers position). The tool was based on a commercial grabber (Unger Enterprise Inc, CT, USA; Fig. 1) with an ergonomic handle fitted with a lever, a long rigid shaft, and a “hand” with two articulated fingers. We customised three different tools, scaling them according to the children’s height. To prevent focusing attention on participants’ arm length, potentially biasing the explicit estimation task, we first determined the ratio between tool length and arm length from the above-cited studies in adults (that is the tool is usually 1.5 times longer than adults’ actual forearm length). Then, we used data from our previous and ongoing studies on children to calculate the relationship between arm length and height in younger participants. Note that also in this study, participants’ height and forearm length strongly correlated (Pearson’s r = 0.89; *p* < 0.001). Finally, we determined the three best tool lengths to fit children on our period of interest. This was to ensure that the manipulated tool would fit the participants’ body size. As a result, the big tool was 40 cm long, similarly to the one we used on previous studies^4–6,8^, except that it was 100 g lighter to prevent fatigue in the younger adolescents; it was used for the young adults and the adolescents who were taller than 147 cm (66 participants; range from 9.2 to 21.5 years old). The middle one, for children between 123 and 146 cm of height, was 32 cm long (23 participants; range from 7.6 to 11.9 years old). The small one was used for the only child under 122 cm (age 7.5) and was 25 cm long. A summary of this can be found in Table 1.

### Gesture imitation proficiency and puberty level assessment

Before or after the kinematic part of the study (randomized), participants performed a modified version of a gesture imitation task, initially developed for the assessment of apraxia^61^: they used their dominant arm to imitate the movement performed by the experimenter using the same arm as the participant (anatomical imitation; note that in the original task, imitation was mirrored). Two examples were first used to familiarize them with the task, and to emphasize the importance of doing the exact same gesture (fingers opening, hand orientation and so on), and that some gestures would be repeated. Instructions were repeated several times before the test. There were 24 items: participants scored 3 points if they imitated correctly the first time, 2 points if the experimenter had to show them the movement a second time. At the third and last repetition, participants had 1 for a correct gesture and 0 for a wrong one. Assessment criteria were similar to those classically used in motor imitation tasks^62^. Specifically, the criteria included the arm or/and hand configuration (extended arm and fist configuration for example), the limb orientation in space (e.g. palm down), its target location (e.g. palm on the contralateral shoulder). For sequential gestures, an additional criterion was the correct order and number of occurrence (e.g. three repetitions of fist and hand flat on the table sequence). Any differing element from one of these criteria was considered as an incorrect imitation. The maximum score was 72. The same, trained experimenter demonstrated the gesture and evaluated online the imitation for all the participants. The rationale behind using this task was to quantify participants’ visuomotor proficiency and to assess whether puberty and growth spurt in particular could affect it, as it has been reported elsewhere^46,51^.

Participants’ height, weight, as well as arm and forearm lengths were measured after the experimental session. Their pubertal development stage was assessed through the Self-Rating Scale for Pubertal Development^63^, an adapted version of a previous interview-based puberty scale^64^. Scores on each of the five items (growth spurt, body hair, skin changes, deepening of the voice/breast growth and growth of hair on the face/menstruations) were added to obtain a global Puberty Score (PS/20). For each item, participants could indicate that the phenomenon had not started yet (hereafter pre puberty), had barely started (early puberty), was definitely underway (mid puberty), or was finished (late puberty). Table 1 summarises, for each puberty score, the participants’ characteristics.

### Kinematic recording system

Similar to previous studies using this paradigm^4–8^, we placed infrared light emitting diodes (IREDs) on three different locations on the participants’ dominant hand: the medial lower corner of the thumb nail, the lateral lower corner of the index finger nail and on the skin proximal to the styloid process of the radius at the wrist. Three more IREDs were located on the tool: on its “fingers” and on the distal part of the shaft (“wrist”). Spatial localization of the markers was recorded with an Optotrak 3020 (Northern Digital Inc; sampling rate: 200 Hz; 3D resolution: 0.01 mm at 2.25 m distance).

### Statistics

We used the same statistical approach for each task: using mixed models, we modelled the changes in the parameters with a fixed factor (PRE/POST or FIRST/LAST BLOCK) and the continuous Puberty Score (see following sections for equations in each model). This allowed to investigate how using a tool could change the kinematic pattern, and how it could do it differently depending on the puberty level. For this last reason, presence of interaction between Puberty Score and the fixed factor was particularly relevant. Since age and puberty are highly correlated and our scope is not to disentangle their respective role, we preferred using the puberty score to have an individual accurate estimation of body growth and indirectly account for the effect of sex. Indeed, the same level of puberty (hence growth spurt) is typically attained at different ages in boys and girls^65^ and within-sex differences have long been known^66^, as confirmed in our dataset; for instance, a boy aged 13.8 years had barely started his puberty (scoring 7/20 on the puberty scale), while a boy aged 13.2 years was in the middle of his growth spurt (scoring 12/20). Due to this complexity, we can expect a non-linear relationship between puberty score and parameters (i.e. the dependent variable). For this reason, we allow the relationship to be quadratic (polynomial of degree 2) or linear.

As we are in a multivariate setting (9 kinematic parameters per task) and each parameter may be associated to a slightly different pattern (i.e. not all the parameters might be significantly affected), we first summarized the 9 kinematic parameters trough an ordination method, namely the Principal Component Analysis for both the free-hand and tool-use movements. We computed the first principal component through a spectral decomposition of the correlation matrix of the 9 parameters (the use of the correlation instead of the covariance matrix is advocated by the need for a principal component that does not depend on the unit of measurement of the parameters). Then the scores of the first principal component is taken as a new meta-parameter (i.e. dependent variable) on the quadratic/linear model for hand/tool movement. By allowing a quadratic polynomial relationship between puberty and the meta-parameter, we add flexibility to the fit, while the linear model uses less degrees of freedom and provides more accurate estimates if the underlying model is truly linear. For each task, we decided between the linear vs. the polynomial through a goodness of fit test (based on the likelihood ratio of the two models). The polynomial/linear fit was used accordingly for all the parameters of the task. The fit of the model for the meta-parameter allows for a multivariate analysis and the significance on a coefficient can be interpret as an overall evaluation among the 9 parameters. We thus additionally distinguished the reaching (latency and amplitude of wrist/tool acceleration, velocity and deceleration) and the grasping component (maximum grip aperture and its latency) and computed a global p-value for each component. We report the percentage of variance explained by the first component of the analysis: higher is this percentage, better the results of the model summarize the multiple parameters (there is no minimum threshold of the captured variance percentage, but we consider that explaining at least 50% of the total variance – from multiple parameters – with a single meta-parameter is a good result).

#### Free-hand movement

The model based on the principal component analysis was better fitted with a quadratic curve than a linear one (χ^2^(2) = 7.28; *p* = 0.026). We thus used a polynomial fit for each kinematic parameter of the free-hand movement. Based on our hypotheses mentioned in the introduction, we analysed the kinematic features of free-hand movements performed after tool use as a function of participants’ adolescence level, as indexed by their puberty score, in order to look for the adult pattern of kinematic modification (increased latencies and reduced peaks). We modelled the data to analyse the fixed effects of session (PRE/POST), with puberty score as a covariate, and their interaction, while taking into consideration the inter- and intra-individual variability. For each parameter, the model equation was the following: parameter ~ session + poly(puberty,2) + session × poly(puberty,2) + (1|subjects). We especially looked for a main effect of session indicating that children, adolescents and young adults had modified kinematics after tool use, as well as an interaction with puberty which would indicate that the pattern changes with development.

#### Tool use

The model based on the principal component analysis was not better fitted with a quadratic curve as compared to a linear one (χ^2^(2) = 3.03; *p* = 0.219). We thus used a linear fit for each kinematic parameter of the tool movement. As the kinematics characteristics of the tool session are known in adults, here we investigated if and when the same patterns were observable in children and adolescents. Adult pattern reports no or very few kinematics differences between the first and last block of tool use, attesting of a reduced/absent motor learning (e.g.^5,8^). Here, in order to search for an effect of development on tool use session we analysed the kinematics differences between the first and fourth block (fixed effect FIRST/LAST) as a function of our continuous variable i.e. the puberty score. For each parameter, the model equation was the following: parameter ~  block  + puberty +  block × puberty + (1|subjects). An absence of difference between the first and last block of tool use would witness an absence of observable training effect. Any interaction would indicate a change in the pattern as a function of pubertal development.

#### Arm length estimation

Previous data in adults showed no influence of tool use^8^. We modelled the Arm Length Estimation task, to analyse the effect of session (PRE/POST), with puberty score as a covariate. Model comparison showed that data was better explained with a polynomial fit than a linear one (χ^2^(2) = 11.26; *p* = 0.004). The model equation was the following: estimation ~ session + poly(puberty,2) + session × poly(puberty,2) + (1|subjects). Absence of tool use effect on this task would be indicated by an absence of main effect of session. Any interaction would indicate a change in the pattern as a function of pubertal development.

Mixed model analyses were performed with “R”^67^ on participants’ individual trials (18 per subject, per PRE/POST session; 12 per subject per block for the tool session) for each kinematic parameter (or for the meta-parameter from the Principal Component Analysis), and on the length estimation (18 trials per subject, per session). We used *lme4*^68^, *lmerTest*^69^ and *car*^70^ packages. The package *car* was used to obtain main effects and interactions from the output of *lme4* (based on Type III Wald chisquare tests; we report these values in the results rather than the output from lme4). This analysis, akin to an ANCOVA in terms of interpretation, allows a better account for variability and for the unbalanced sample size for each puberty score.

Using this method, in case of significant interactions indicating a change in the kinematic pattern, we can also calculate the exact point where this occurs, for each kinematic parameter. To do so, from each fitted model we estimated the (non-linear) curve of the parameter (i.e., Velocity, Acceleration etc.) for each condition. We tested point-by-point whenever the two conditions differ. The interval of Puberty Scores where differences between PRE and POST were not significant (significance level 5%) is defined as the interval of equivalence. The crossing point of the curves of the two conditions is the point where the two conditions equalise. We report both crossing points and intervals of equivalence. We additionally computed the crossing point on the meta-parameter from the principal component analysis. We prefer this method since it allows to compute the global interval of equivalence, which is not possible when simply reporting the average and median of each individual crossing point. For sake of completeness, we still report these values in the analysis.

## Results

### Free-hand movement

#### Tool use affects the reaching kinematics differently according to puberty

Systematic main effects of session (PRE/POST) were observed on the reaching component for all the amplitudes (all *p* < 0.004), and on the acceleration latency (χ^2^(1) = 6.61; *p* = 0.010). No significant session effect was observed on the velocity latency (χ^2^(1) = 0.035; *p* = 0.851), the deceleration latency (χ^2^(1) = 0.733; *p* = 0.392) and movement time (χ^2^(1) = 0.266; *p* = 0.606). These results indicate a global effect of tool use on subsequent free-hand reaching movements, which will be interpreted in line of the presence or absence of an interaction. Conversely, there was no main effect of puberty score on any of these parameters (all *p* > 0.441) or movement time (χ^2^(2) = 0.615; *p* = 0.735).

**Figure 2:**
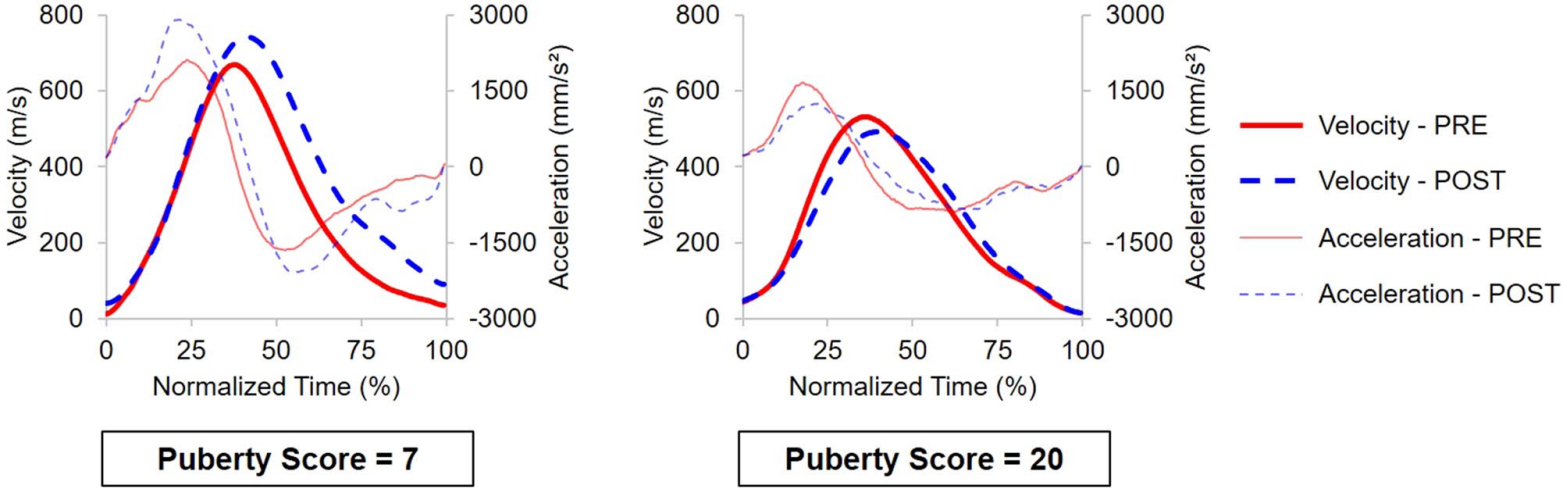
Average kinematic profile (18 trials) of representative early puberty (PS = 7, left panel) and late puberty participants (PS = 20, right panel). The kinematics changes after tool use are opposite as a function of the puberty score. Instead of the increased latencies and reduced amplitudes displayed by late puberty participants, early puberty ones displayed shortened latencies and larger peak amplitudes.

**Figure 3:**
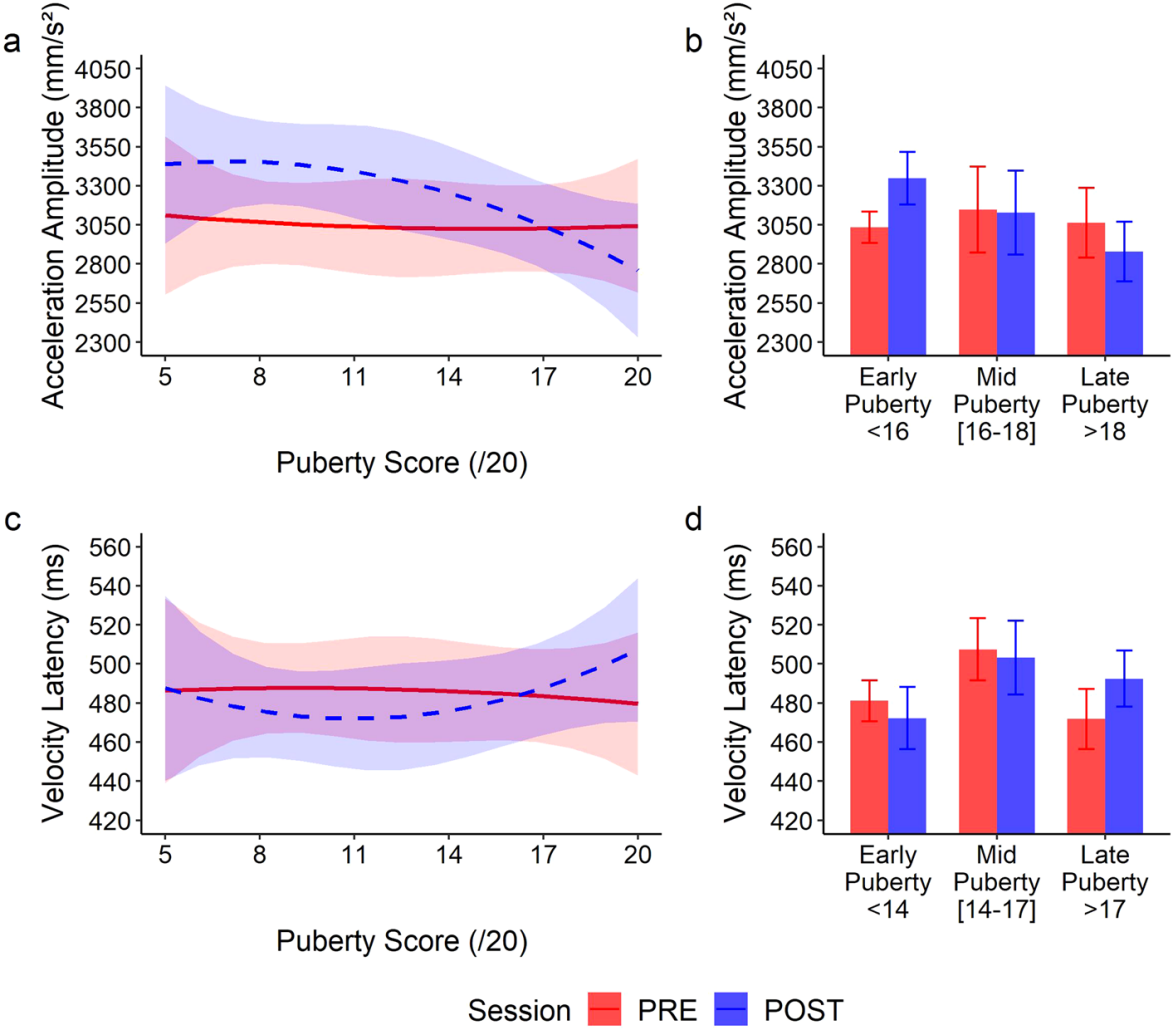
Evolution of the kinematics of the Reaching component of the movement before (solid red) and after (dashed blue) tool use, as a function of the puberty score. Panels (**a**) and (**c**) are representative illustrations of the polynomial fit of the data for the acceleration amplitude (**a**) and the velocity latency (**c**), with the 95% confidence interval indicated by colored areas. Puberty score did not affect the initial movement kinematics (PRE: solid red) but changed participants’ behavior after tool use (POST: dashed blue) as shown by significant interactions. (**a**) After tool-use early puberty participants displayed higher acceleration amplitude (blue line with respect to the red line); beyond mid puberty, participants reversed their kinematics patterns and displayed smaller peaks after tool use. (**c**) A pattern reversal was also observed for the latency of the wrist velocity, though opposite. For better visualization, panels (**b**) and (**d**) report three groups we computed for the same parameters based on the puberty score, the mid-puberty group being the one for which no significant modification was observed between PRE and POST in the model (cut-off scores differing for each parameter, see section “Crossing Point” below). The typical adult pattern (reduced peaks and increased latencies) emerges only in the late puberty stage, after the crossing point in the model. Error bars indicate the means ± 1 sem.

Of main interest, significant interactions between session and puberty score were observed on all parameters (all *p* < 0.001), except the acceleration latency (χ^2^(2) = 5.27; *p* = 0.072). While reaching kinematics was initially independent of the puberty score, tool use-dependent changes were highly modulated by puberty stages (Fig. 2). Indeed, after tool use, early-puberty participants displayed larger amplitudes (Figs. 2 and 3a,b) and shorter latencies (Figs. 2 and 3c,d). They reached faster with their hand after having used the tool. Conversely, after tool use, late-puberty participants displayed smaller amplitudes and longer latencies, resulting in a longer movement time (Figs. 2 and 3).

Consistency of these results on the whole reaching component were confirmed by the principal component analysis (percentage of explained variance for the first component: 66.2%), with no significant effect of the puberty (χ^2^(2) = 0.22; *p* = 0.898), a significant main effect of session (χ^2^(1) = 7.14; *p* = 0.008) and a significant global interaction between puberty and session (χ^2^(2) = 58.1; *p* < 0.001). Overall, puberty did not impact the initial free-hand kinematics but did affect the pattern observed after tool use.

#### Tool use affects the grasping kinematics according to puberty

**Figure 4:**
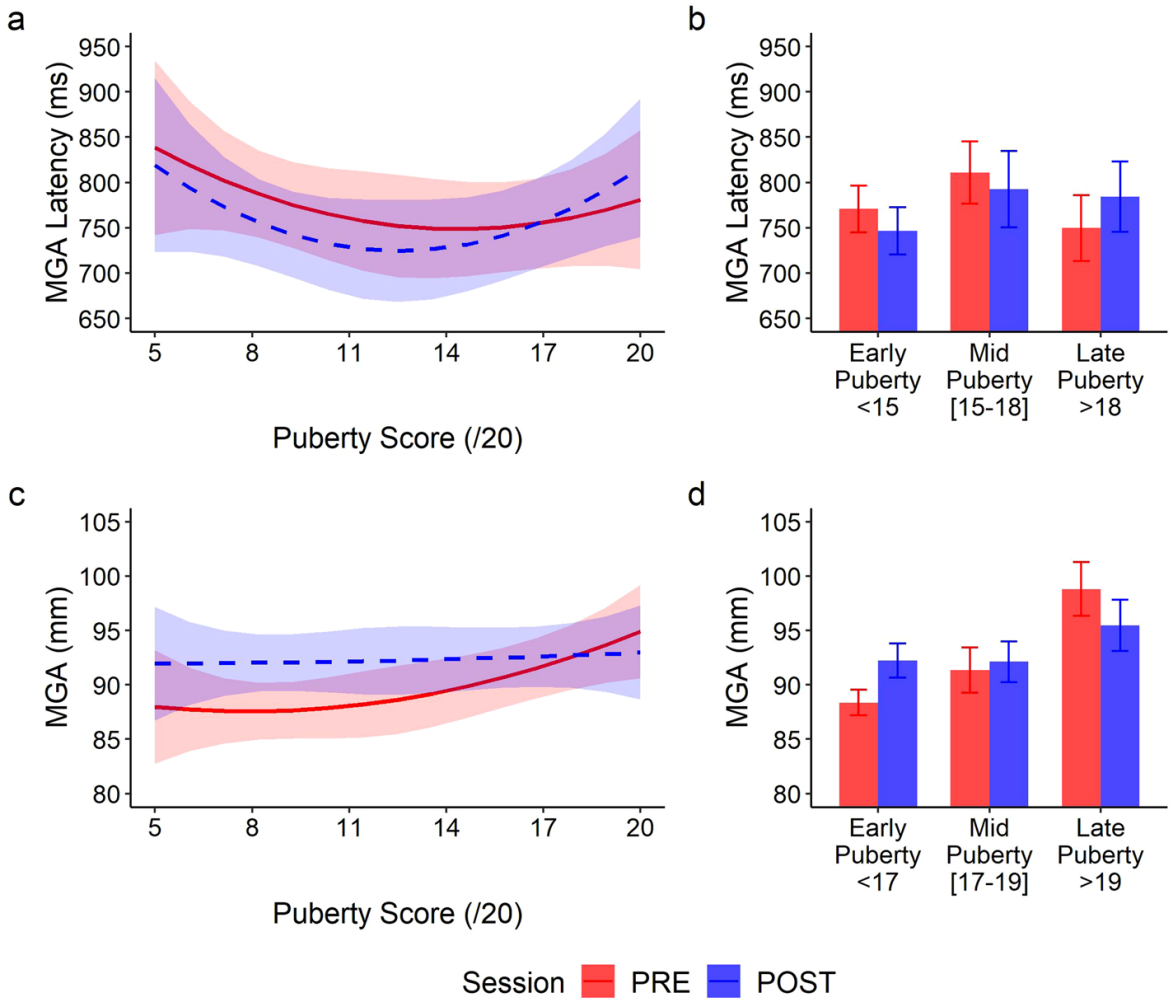
Evolution of the kinematics of the Grasping component of the movement before (solid red) and after (dashed blue) tool use, as a function of the puberty score. Panels a and c are representative illustrations of the polynomial fit of the data for the Maximum Grip Aperture latency (**a**) and its amplitude (**c**), with the 95% confidence interval indicated by colored areas. (**a**) After tool-use early puberty participants displayed shorter MGA latency (blue line with respect to the red line); beyond mid puberty, participants reversed their kinematics patterns and displayed longer latencies after tool use. (**c**) A pattern reversal was also observed for the finger aperture, with less difference with development. For better visualization, panels (**b**) and (**d**) report three groups we computed for the same parameters based on the puberty score, the mid-puberty group being the one for which no significant modification was observed between PRE and POST in the model (cut-off scores differing for each parameter, see section “Crossing Point” below). Here we clearly see that differences between PRE and POST are still present in the late adolescent group, at odds with what is usually observed in adults. Error bars indicate the means ± 1 sem.

Similar effects were observed on the grasping component with significant main effects of session (PRE/POST) for the MGA latency (χ^2^(1) = 4.75; *p* = 0.029) and the MGA (χ^2^(1) = 56.1; *p* < 0.001). A significant main effect of puberty was observed for the MGA peak (χ^2^(2) = 7.35; *p* = 0.025), but not for its latency (χ^2^(2) = 1.88; *p* = 0.391). Interactions reached significance for both parameters (all *p* < 0.001; Fig. 4). Regarding latency, this interaction was similar to what reported above: early puberty participants opened their fingers sooner after tool use, while late puberty ones displayed longer latencies (Fig. 4a,b). Concerning the grip amplitude, the significant interaction with puberty suggests that grip aperture was more dependent on puberty before tool use. In the PRE session, late puberty participants displayed larger grip aperture. Yet, after tool use, grip aperture did not vary as a function of puberty (Fig. 4c,d). Noteworthy, late puberty participants still modulated their grip aperture after tool use.

Consistency of these results on the whole grasping component were confirmed by the principal component analysis (percentage of explained variance for the first component: 62.3%), with no significant effect of puberty (χ^2^(2) = 4.51; *p* = 0.11), a significant main effect of session (χ^2^(1) = 34.9; *p* < 0.001) and a significant global interaction between puberty and session (χ^2^(2) = 58.2; *p* < 0.001). Overall, puberty did not impact the initial kinematics, but affected the pattern observed after tool use.

#### Crossing point

For each parameter of the reaching and grasping components, we observed a crossing point on the puberty scale beyond which the kinematics pattern reversed. Table 2 reports this crossing point and its interval of equivalence (no significant difference between PRE and POST) for each parameter, as given by the Principal Component Analysis.

**Table 2:** Point and area around where there was no difference between PRE and POST, and after which a change in kinematic pattern occurred.

Kinematic parameters	Crossing point (/20)	Interval of equivalence (i.e. area of non-significance around the mean; full range is [5; 20])
Acceleration latency	12.45	[5; 17]
Acceleration amplitude	17.23	[16; 18]
Velocity latency	16.24	[14; 17]
Velocity amplitude	17.54	[16; 19]
Deceleration latency	14.29	[11; 17]
Deceleration amplitude	16.71	[15; 18]
MGA latency	16.71	[15; 18]
Maximum grip aperture	18.02	[17; 19]
Movement time	12.15	[10; 15]
**Overall**		
Average	15.7	X
Median	16.7	X
Meta-parameter (from the PCA)	16.0	[14; 17]

PCA stands for Principal Component Analysis and indicates the overall crossing point when grouping all parameters, with its interval of equivalence. Puberty score ranges from 5 to 20.

After grouping all the crossing points together, a Principal Component Analysis revealed that average crossing point was 16 on the puberty scale, with an interval of equivalence of [14; 17]. This result is also consistent with the average (15.7) and the median (16.7) crossing point among all parameters. When referring to the puberty scale^63^, such a score corresponds to participants getting a score of about 3 for each question meaning that changes are “definitely underway”, as defined in the scale, which corresponds to mid puberty.

### Tool movement

#### Tool use practice effects on reaching reduce with puberty

Systematic main effects of block (FIRST/LAST) were observed for all the parameters of the reaching component and the movement time (all *p* < 0.026). This indicates a global improvement in performance on the last block of tool use: participants were faster at the 4th block of practice with the tool, resulting in a shorter movement time. Conversely, there was no effect of puberty score on any of the parameters (all *p* > 0.439, except Movement Time: χ^2^(1) = 3.34; *p* = 0.067).

**Figure 5:**
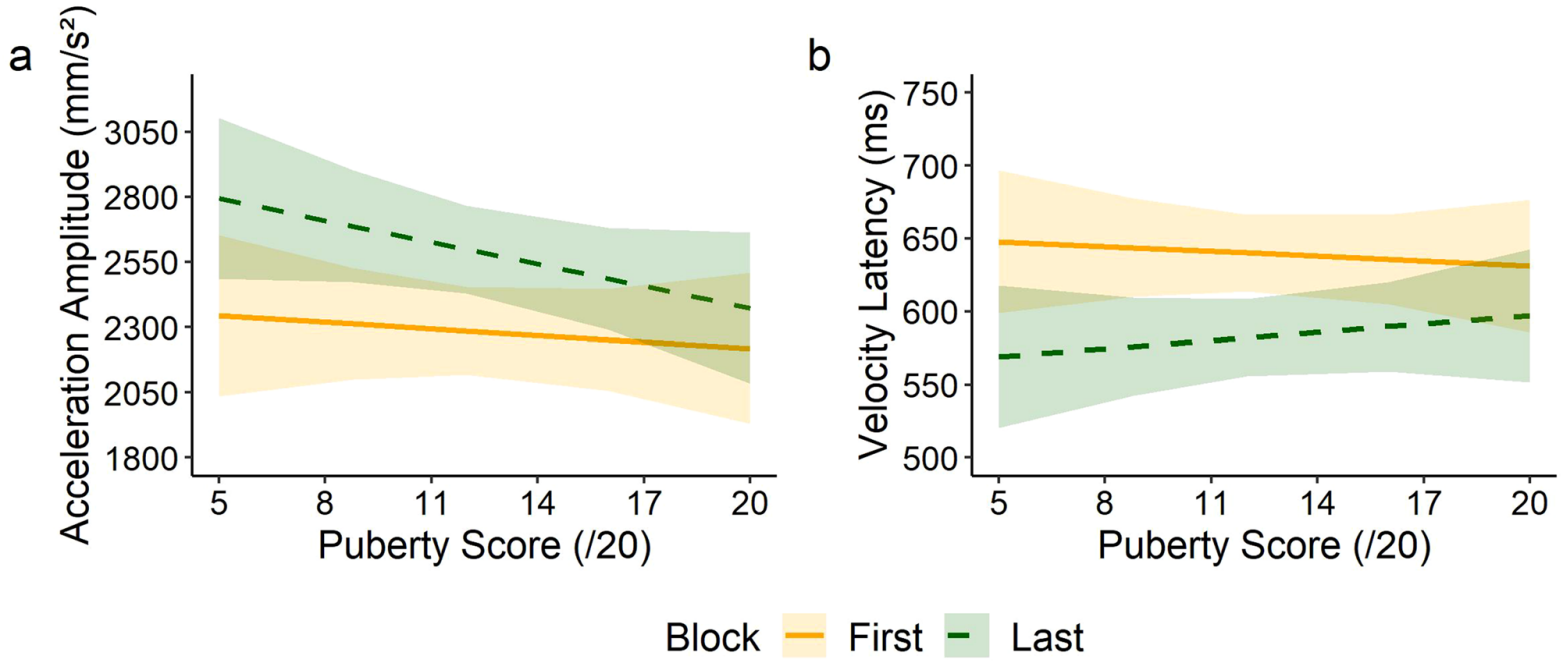
Evolution of the kinematics of the Reaching component of the movement during the first (solid orange) and the last (dashed green) blocks of tool use, as a function of the puberty score. Panels a and b are representative illustrations of the polynomial fit of the data for the acceleration amplitude (**a**) and the velocity latency (**b**), with the 95% confidence interval indicated by colored areas. Most significant interactions indicated that improvement through tool use is less important in late puberty.

Significant interactions were observed in 3 out of 6 reaching parameters: acceleration latency (χ^2^(1) = 5.17; *p* = 0.023), acceleration amplitude (χ^2^(1) = 11.5; *p* < 0.001) and velocity latency (χ^2^(1) = 7.67; *p* = 0.006). Interaction with movement time was also significant (χ^2^(1) = 26.8; *p* < 0.001). These interactions indicate that the difference between the first and the last block decreases as the puberty score increases (Fig. 5a,b).

Consistency of these results on the whole reaching component were confirmed by the principal component analysis (percentage of explained variance for the first component: 60.9%), with no significant effect of the puberty (χ^2^(1) = 0.02; *p* = 0.879), a significant main effect of block (χ^2^(1) = 57.3; *p* < 0.001) and a significant global interaction between puberty and block (χ^2^(1) = 9.58; *p* = 0.002). Overall, puberty did not impact the kinematics during tool use and training effects reduced as puberty progresses.

#### Tool use practice effects on grasping change with puberty

There was a main effect of block on MGA latency (χ^2^(1) = 18.5; *p* < 0.001), but no effect of puberty score (χ^2^(1) = 0.646; *p* = 0.422), nor an interaction between the two (χ^2^(1) = 1.07; *p* = 0.300). This indicated that all the participants, whatever their puberty score, opened the tool “fingers” earlier in the last block of tool use, consistent with a faster movement. As for the MGA, there was a main effect of block (χ^2^(1) = 12.6; *p* < 0.001), of puberty (χ^2^(1) = 8.28; *p* = 0.004), and an interaction between the two (χ^2^(1) = 23.3; *p* < 0.001). This indicated that late puberty participants displayed a larger opening of the tool and reduced their MGA during the last block of tool use, while younger ones did not particularly modulate it.

Consistency of these results on the whole grasping component were confirmed by the principal component analysis (percentage of explained variance for the first component: 60.4%), with no significant effect of the puberty (χ^2^(1) = 1.99; *p* = 0.158), a significant main effect of block (χ^2^(1) = 22.9; *p* < 0.001) and a significant global interaction between puberty and block (χ^2^(1) = 10.56; *p* = 0.001).

### Arm length estimation

**Figure 6:**
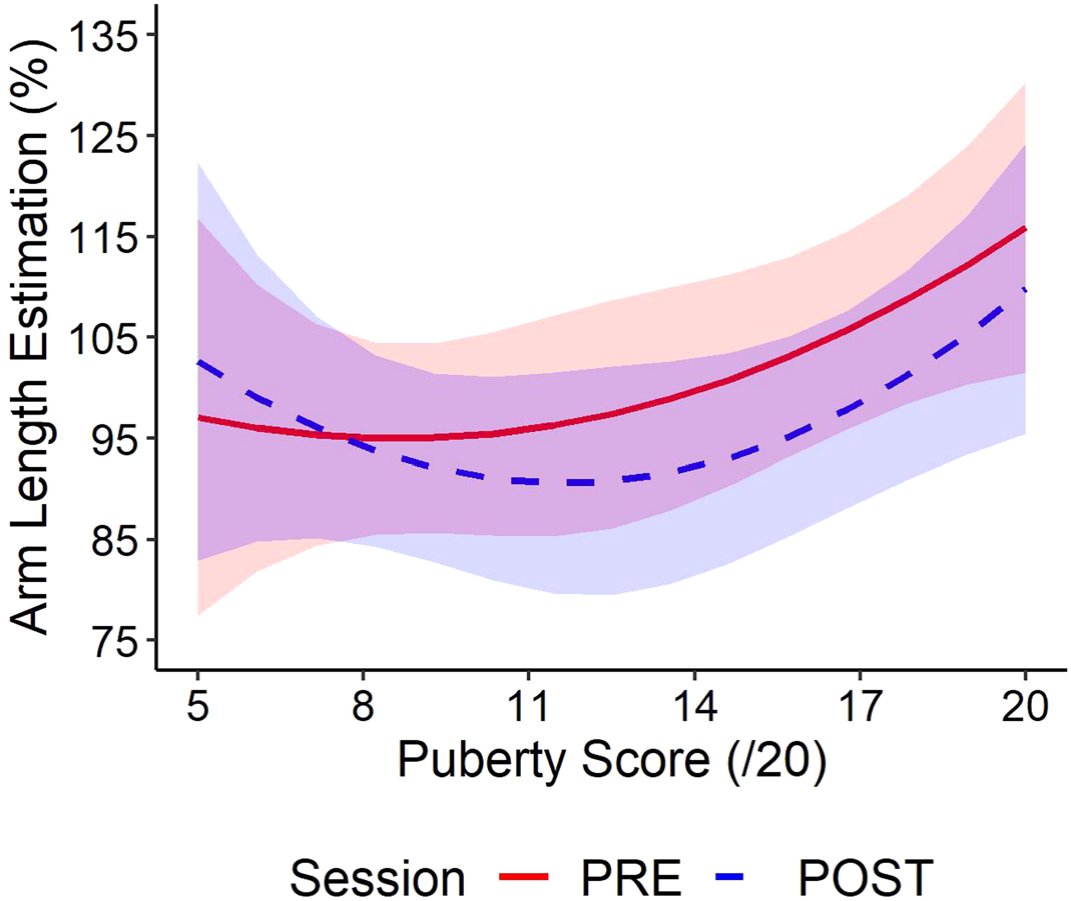
Arm length estimation in percent of actual arm length as a function of the puberty score. Most participants (puberty score > 9) estimated their arm as shorter after tool use.

As stated earlier, this task allowed to control for the specificity of tool use on different body representations. As it is an explicit judgement of arm length, this tasks targets the conscious body image, so far reported as being immune to tool use in mid-twenties adults^8^. Transforming the estimation in percent of the veridical arm length allowed us to assess the effect of puberty independent of the difference in arm length over development. There was a main effect of the session (χ^2^(1) = 42.6; *p* < 0.001) showing a reduced estimated arm length after tool use, a non-significant effect of the puberty (χ^2^(2) = 5.04; *p* = 0.080) and an interaction between the two (χ^2^(2) = 35.6; *p* < 0.001). The non-significant effect of puberty suggests that overall, participants did not estimate their arm length differently according to their pubertal development. The interaction highlighted a very early crossing point (7.5), with no significant differences between PRE and POST for puberty scores [6; 8]. It thus appears that most of the participants (puberty score > 9) modulated their estimation after tool use in the direction of a shorter arm after tool use, while early puberty participants did not. (Fig. 6).

### Gesture imitation proficiency

**Figure 7:**
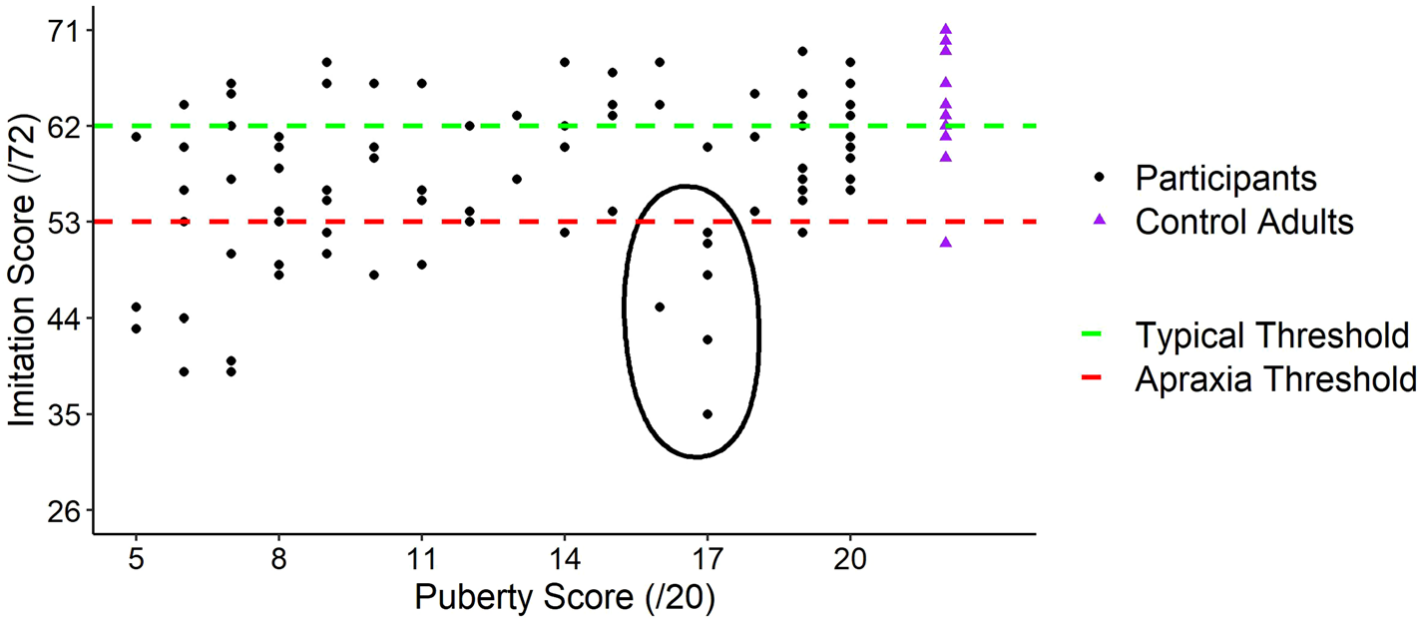
Individual scores on the gesture imitation test. Globally, performance increased with puberty. Yet, most mid puberty participants displayed poor performance, as indicated by the black ellipse. This group had a mean puberty score around the crossing point (see “Crossing point” section).

As some studies reported that puberty could lead to transient clumsiness^46,51^, we measured the gesture imitation abilities of our participants as an index of such clumsiness. The total mean on gesture imitation task was 57/72 ± 7.7 (see Table 1 for details per puberty score). Performing the imitation anatomically increased task difficulty, as the participants’ score was overall lower than the cutoffs values when imitation is mirrored as in the original version (Fig. 7; healthy ≥ 62; apraxia ≤ 53^61^), for most of the sample as well as an additional group of 12 older healthy adults that we recruited (Mean age = 26.2 ± 2.5; 7 girls; three left-handed; Mean score = 63/72). These categorizations should thus be here considered comparatively (across pubertal stages).

When running simple regression, it appeared that a linear model was the best fit compared to any quadratic one: the more mature, the better the gestures reproduction. However, when looking at individual performance, participants matching the puberty crossing point stood out (black ellipse; Fig. 7), as they showed a decrease in performance.

## Discussion

In this study, we investigated whether, when and how tool-use-induced plasticity of body representation, typically observed in mid-twenties adults (for review, see^11^), emerges and evolves during development. In particular, we tested whether any changes in free-hand kinematics are observable during the abrupt variation introduced by the growth spurt in adolescence. We asked typically developing participants at different level of their puberty to reach and grasp an object with their dominant hand both before and after using a tool to perform the same task. To control for the specificity of the effects on the body representation for action, we further tested the same participants for their explicit, subjective estimate of their arm length^8^. Besides serving as a control for the effects of tool use on the implicit body estimate, this task is one among those that can inform about possible effects of tool use on another, explicit and conscious body representation, namely the body image^11^.

Regarding the implicit arm representation, results showed that tool incorporation, as witnessed by kinematics, seems to evolve importantly during development and requires a previously unsuspected long time to manifest. At the earliest puberty stages, tool incorporation displays a pattern opposite to the one previously documented in adults, as if arm length representation shrank after tool use. Then it progressively reverses, to reach the adult-like pattern only once puberty is finished, through a period in the middle of puberty when changes in kinematics are literally absent. Interestingly, and also at odds with what reported in adult tool users^5,6^, most participants’ performance showed training effects during the tool-use phase: tool movements performed during the last block of trials were faster than those performed during the first block of trials. Maturation erases the differences between the first and last tool use blocks and progressively settle the increase in arm length representation classically observed after tool use in adults.

Regarding the explicit arm length estimation task, again contrasting what was previously reported in adults^8^, most of the participants estimated their forearm as shorter after tool use. In adults, indeed, the visually-driven representation of the body (also called body image) is largely immune to tool use effects, as measured by this task^6,8^. Yet, here, we found that this subjective estimate of the arm length was also affected by tool use, and until the late pubertal stages. Two possibilities may account for this difference. On the one hand, as discussed below, vision may dominate during development for achieving tool control. This major use of visual information for movement guidance could blur the difference between body state and body image. On the other hand, when no effects on the arm length estimation task were observed after tool use^8^, participants were blindfolded while performing the tool use session. The absence of visual feedback during tool use may thus have facilitated the distinction between these body representations. Interestingly, there was no crossing point around mid-puberty in this task (by opposition to the free-hand reach-to-grasp task). This suggests that changes occurring during growth spurt are likely to play a minimum role in the conscious perception of the arm length. Overall, the availability of visual feedback may be at the core of the explicit arm-length underestimation we report here after tool use.

### Tool use effects in early puberty: a visually-mediated decrease in arm-length representation?

Early-puberty participants showed a kinematic pattern opposite to that reported by previous studies in adults. In addition, they showed kinematics changes during the tool-use session: they were faster during the last block of tool use, as compared to the first one. After tool use, the pattern of reduced latencies and higher peaks is suggestive of an update of the length of the effector estimate, which is compatible with an arm that is represented as shorter. Indeed, early puberty was accompanied by a performance that is opposite to that documented in adults (increased latencies and smaller peaks), which is considered as a lengthening or their arm representation (see, for review^11^). At first sight, the possibility that tool use shortens arm representation may seem unlikely: since the body cannot biologically shrink, it has been posited that the representation the brain used to control it can only accept increases in size in healthy individuals^5,71–73^. However, a recent study whereby arm length representation has been tested during tool motor control, reported an initial fast reduction of the arm length representation, followed by its (expected) increase after protracted tool use^74^. Ganesh and colleagues^74^ explained the subsequent increase in body representation dimension as a result of the construction of new sensorimotor associations. These would serve reducing the computational time and cost for motor planning with the tool, hence guaranteeing its skilful use. Conversely, the immediate reduction in arm length representation would follow from the need of using a new tool, for which no sensorimotor association has yet been built. The control over this new tool would entail a safety margin, enabling to perform the reach under visual feedback guidance^74^. Accordingly, the decrease in arm representation would depend on the use of visual feedback. Following this account, the early developmental stages would allow to progressively build sensorimotor associations when facing new tools. In our study, participants used this tool for the first time, the difference between the first and fourth block of tool use might then reflect the building of new, more appropriate sensorimotor associations. A previously used tool shall conversely call for the recruitment of old sensorimotor associations and neither training nor decrease in arm length representation may take place. As a non-exclusive alternative, an immature proprioceptive processing, might urge the use of visual information^75,76^. As recalled in the introduction, the integration of proprioceptive information is crucial for body representation. In movement execution, children around 7–8 years old experience a transient difficulty in using proprioceptive feedback^43^ and improvement in proprioceptive processing would significantly improve sensorimotor integration, suggesting that proprioceptive integration grows with development^41^. When both visual and proprioceptive input allow localization of the target/or participant’s own hand, early puberty participants favor visual information^37,38,41,77,78^. This may result in a protracted development of body representations, children not representing their body size truthfully until quite late^33^.

### Tool use effects in late puberty: an adult-like, but still incomplete pattern

Our findings on free-hand movements after tool use highlight the existence of a developmental “threshold” after which the impact of tool use on free-hand kinematics resembles that of adults. Such threshold wherein adolescents were in the middle of their puberty and growth peak, was marked by the absence of any kinematic modifications of free-hand movements after tool use. While this suggests the absence of tool use plasticity in the representation of their motor effector, the difference in tool kinematics between tool use blocks suggest an on-going building of sensorimotor associations. Beyond this point, participants displayed the kinematic profile of adult-like tool-use plasticity (longer latencies and smaller peaks), which constitutes a clear pattern reversal. Yet, their motor control was different from what has been previously observed in adults, in two main respects. First, despite being drastically reduced as compared to earlier pubertal stages, kinematic modifications were still at play during tool use on some parameters, thus indicating a not fully mature process. Indeed, no change in any of these parameters has ever been observed in previous work in adult participants^5,6,8^. Second, the changes after tool use affected both the reaching and the grasping components of their movements. Following the use of the type of tool we employed in this study, which mainly elongates the arm functionality, adults’ kinematic modifications are typically restricted to the transport component of the movement^5,6,8^. These effects are indeed mainly driven by the tool functional features^72,79,80^.

In this respect, though more adult-like, the pattern of the late puberty participants was still incomplete. Noteworthy, similar findings have been previously reported in the case of a deafferented adult patient^59^. Following a medullar lesion, patient DC was still capable of perceiving superficial touch on her affected arm or on a hand held tool^81^, but had lost proprioception and her tool motor control was profoundly altered. After repeated sessions of tool use, her free-hand kinematics tended to normalize. Indeed, in adults, motor learning is possible in absence of proprioception^82,83^, further suggesting that vision might be predominant in the case of tool use for the early pubertal stages of development. Two elements characterized the profile exhibited by the deafferented patient, though. Akin to what observed in late puberty participants, both the reaching and grasping components displayed longer latencies and smaller peaks; moreover patient DC displayed tool use training effects, with performance improvement between the first and the last tool use blocks^59^. Thus, the findings from late puberty participants reported here remarkably resemble those of the proprioceptively deafferented adult, in that they both lack of specificity for the transport component and display kinematic modifications during tool use.

We suggest that a slow process of progressively increased reliance on proprioception during development, could be at the basis of the pattern we observed. When their puberty is “definitely underway”^63^, matching the fast growth period^84^, mid-puberty adolescents would still be unable to properly use the proprioceptive signals to update their body state. With the progressive refinement of proprioception, late puberty adolescents would then display a more adult-like behaviour. These findings are in keeping with the transient period of “proprioceptive neglect” during adolescence put forward for postural control, when adolescents fail to make a proper use of proprioception^35,57,58,85^. Note that, when the use of visual feedback is sufficient to fulfil a task, adolescents do not behave as if their body representation could not follow their growth spurt^47^, reinforcing the central role played by proprioception. Interestingly, the crossing point in which no effects of tool use were observed on kinematics, corresponds to puberty scores of adolescents who displayed poor performance in our gesture imitation task. This finding suggests that clumsiness might emerge when body representation plasticity is lacking in either direction^48^. Altogether, these findings suggest that proprioception might be at the core of developmental tool use incorporation and clearly disclose that its contribution goes through numerous changes during development, whereby vision may initially play a greater role and proprioception may later play a greater role at later stages.

In the light of the present findings, we additionally suggest that adults quickly update their body representation and set the state of the effector as ‘longer’ while using a tool, but this plasticity requires a long developmental phase to be achieved in full. During development, novel sensorimotor associations patterns are experienced that may contribute to the ultimate goal of achieving optimal control over novel effectors^8^. This seems to imply (1) to switch the initial vision-based control in favor of a proprioception-based control and (2) to build new sensorimotor associations. Until these processes are not complete, the control of a tool would initially imply to reduce arm length (likely because of the predominant visual control) and the repetitive use of the tool would speed up movement execution (possibly because of a learning mechanism). From this perspective, the developmental trajectory through pubertal stages could replicate what has been observed (in a much shorter, individual time scale) during tool use by Ganesh and colleagues in adults^74^. This may have implications for sports and tool-related skilfulness acquisition (see^46^), suggesting that training might benefit being adapted according to the pubertal stage of the trainees, an interesting question for future research.

## Conclusion

In conclusion, this study discloses that plasticity of body representation following tool use develops until the adult age. Tool incorporation, as indexed by the adult typical kinematic pattern, develops very slowly, becoming apparent only after adolescence. As long as adulthood is not reached, tool use behavior would rather rely on vision, inducing a representational shrinking of the arm after tool use. As they grow up, adolescents would progressively rely on proprioception, before being able to incorporate tool as adults do.

## Data Availability

All data and code used in this study are available on the website of the Open Science Framework (OSF) and can be accessed at https://osf.io/3g9mz/.
